# *In Situ* Vaccination as a Strategy to Modulate the Immune Microenvironment of Hepatocellular Carcinoma

**DOI:** 10.3389/fimmu.2021.650486

**Published:** 2021-05-07

**Authors:** Isabella Lurje, Wiebke Werner, Raphael Mohr, Christoph Roderburg, Frank Tacke, Linda Hammerich

**Affiliations:** ^1^ Department of Hepatology and Gastroenterology, Charité University Medicine Berlin, Berlin, Germany; ^2^ Clinic for Gastroenterology, Hepatology and Infectious Diseases, University Hospital Düsseldorf, Medical Faculty of Heinrich Heine University Düsseldorf, Düsseldorf, Germany

**Keywords:** hepatocellular carcinoma (HCC), immunotherapy, *in situ* vaccine, dendritic cells (DC), tumor microenvironment

## Abstract

Hepatocellular Carcinoma (HCC) is a highly prevalent malignancy that develops in patients with chronic liver diseases and dysregulated systemic and hepatic immunity. The tumor microenvironment (TME) contains tumor-associated macrophages (TAM), cancer-associated fibroblasts (CAF), regulatory T cells (Treg) and myeloid-derived suppressor cells (MDSC) and is central to mediating immune evasion and resistance to therapy. The interplay between these cells types often leads to insufficient antigen presentation, preventing effective anti-tumor immune responses. *In situ* vaccines harness the tumor as the source of antigens and implement sequential immunomodulation to generate systemic and lasting antitumor immunity. Thus, *in situ* vaccines hold the promise to induce a switch from an immunosuppressive environment where HCC cells evade antigen presentation and suppress T cell responses towards an immunostimulatory environment enriched for activated cytotoxic cells. Pivotal steps of *in situ* vaccination include the induction of immunogenic cell death of tumor cells, a recruitment of antigen-presenting cells with a focus on dendritic cells, their loading and maturation and a subsequent cross-priming of CD8+ T cells to ensure cytotoxic activity against tumor cells. Several *in situ* vaccine approaches have been suggested, with vaccine regimens including oncolytic viruses, Flt3L, GM-CSF and TLR agonists. Moreover, combinations with checkpoint inhibitors have been suggested in HCC and other tumor entities. This review will give an overview of various *in situ* vaccine strategies for HCC, highlighting the potentials and pitfalls of *in situ* vaccines to treat liver cancer.

## Introduction

Liver cancer is the fourth leading cause of death worldwide, causing almost 800,000 deaths annually (1, 2). Hepatocellular carcinoma (HCC) is the most frequent primary liver malignancy, accounting for approximately 80% of primary liver cancers (3). The most common etiology of HCC is chronic liver disease, caused by viral infection, alcohol-related liver disease (ALD) and nonalcoholic steatohepatitis (NASH) (4, 5). The overall prognosis of patients with HCC remains poor, despite the establishment of screening programs, advancements in surgical and interventional therapies as well as systemic treatment options (1, 6–8).

Only a small fraction of patients is diagnosed at disease stages still amenable to curative therapies such as orthotopic liver transplantation, liver resection and interventional ablation (9, 10). In intermediate and advanced tumor stages, the majority of patients receive palliative treatment, including interventional strategies, as well as systemic pharmaceutical therapies. The latter have been shaped considerably over the last years, mainly through the discovery of multikinase inhibitors such as Sorafenib in 2008, which was the first drug to improve the survival of HCC patients, however, prolonging overall survival (OS) by less than three months (11). Since then, several other multikinase inhibitors like Lenvantinib, Regorafenib and Cabozantinib gained regulatory approval, however, also showing only modest improvement of patient survival. Immunotherapies such as checkpoint inhibitors represent the most important breakthrough in cancer therapy in the past two decades and were also explored for therapy of advanced HCC (12). However, the response rates to immune checkpoint inhibition as a monotherapy [e.g. Nivolumab, anti-programmed death (PD)-1] in HCC were still very low (about 15-20%), and strongly dependent on the tumor immune status (13, 14). In this regard, defective antigen cross-presentation by dendritic cells (DC), the most important professional antigen-presenting cells, and an exhaustion of the cytotoxic T cell response promote tolerance to the tumor and resistance to checkpoint inhibition (15). Thus, strategies activating the DC-CD8+ T cell axis to restore a CD8+ antitumor response have the potential to improve patients’ outcomes and are intensely investigated. First evidence that combination therapies can improve response to checkpoint blockade in HCC has been provided by a phase III study investigating the combination of the checkpoint inhibitor atezolizumab (anti-programmed death ligand (PD-L) 1) and bevazicumab [anti-vascular endothelial growth factor (VEGF)], which reported outcomes superior to Sorafenib in HCC (12).

Cancer vaccines have been proposed as a strategy to induce or reactivate antitumor immune responses (16). Their mechanism is based on isolating patient-derived DCs, pulsing them with tumor-associated antigens (TAAs) and maturation signals, followed by their reinfusion (17). However, the great variability of tumor antigens and the lack of universal TAAs have prevented their clinical use until now (18). Moreover, the inherent logistical difficulties of preparing individualized vaccines *ex vivo* limits their application. Similarly, T-cell transfer of CD8+ T cells is associated with a simultaneous homeostatic inhibition of T cells, yielding overall disappointing clinical results in solid tumors like HCC (19, 20).

**Figure 1:**
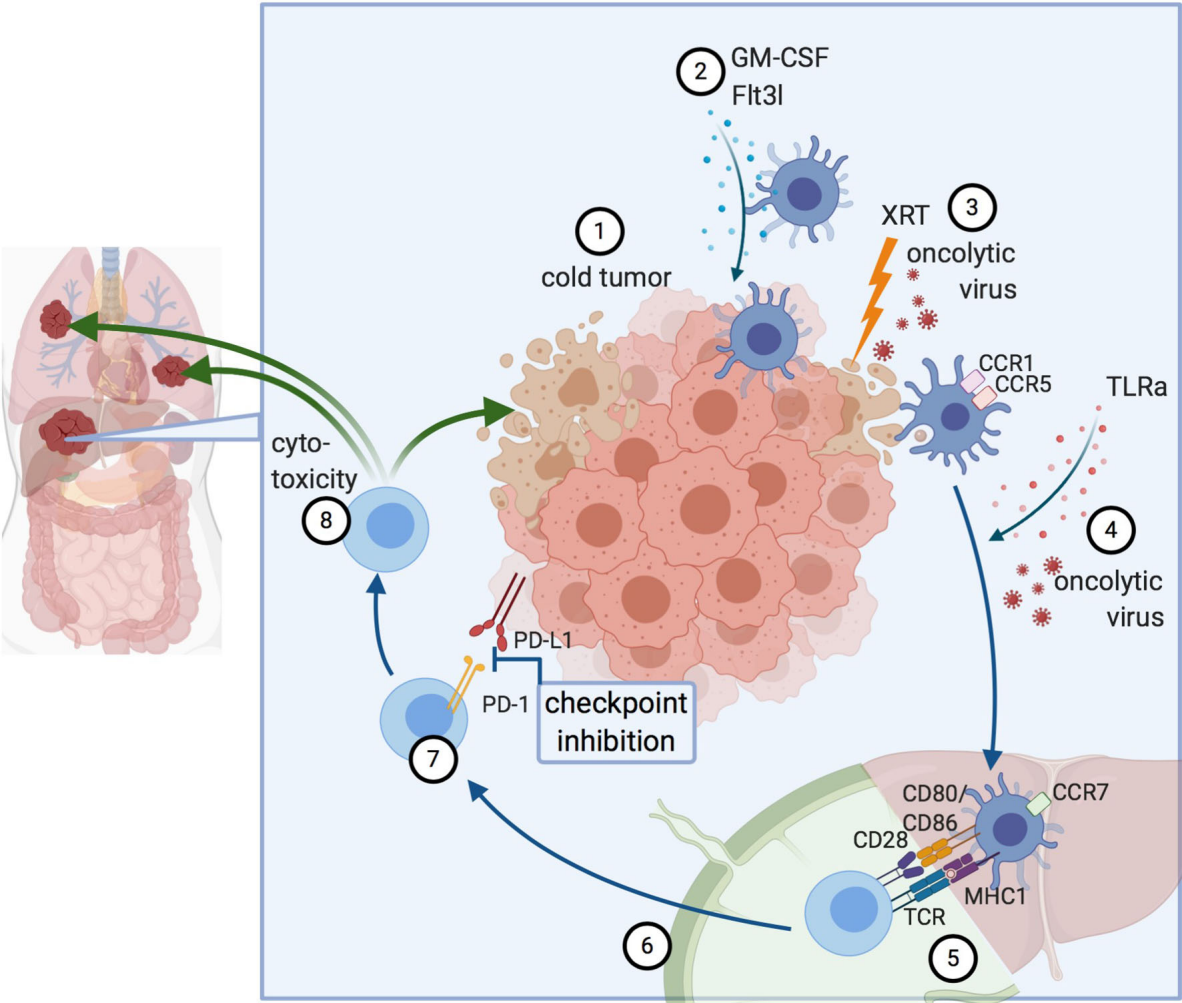
Principles of *in situ* vaccines. 1) Cold tumor devoid of DCs and T cells. 2) DC recruitment to the tumor. 3) Induction of immunogenic cell death, for example by radiation or oncolytic viruses. 4) Maturation signals for DCs lead to 5) Antigen presentation and cross-priming of CD8+ T cells. This can occur either in the liver itself (in tertiary lymphoid organs forming near liver tumors) or the draining lymph node. 6) Activated T cells migrate to the tumor. 7) Abrogation of inhibitory signaling e.g. *via* checkpoint inhibition. 8) Cytotoxicity against the treated tumor and by abscopal effects against other lesions, as well. Created with biorender.

Inducing and stimulating an immune response specifically at the tumor site is referred to as *in situ* vaccine, a concept that takes advantage of the whole repertoire of TAAs available at the tumor site (21). Thus, the intratumoral or systemic injection of immunomodulators can induce presentation of TAAs by antigen-presenting cells (APCs) and, subsequently, the activation of a cytotoxic T cell response with the generation of both effector and memory CD8+ T cells. Several prerequisites for a successful antitumor immune response have been identified: i) The availability of TAAs in a sufficiently immunogenic setting to trigger phagocytosis and activate DCs; ii) an efficient antigen presentation with co-stimulatory signals to successfully cross-prime CD8+ cytotoxic T cells; and iii) a cytotoxic T cell response that overcomes inhibitory signals from the tumor and TME. Collectively, this will result in adaptive antitumor responses with local and systemic effects (**Figure 1**) (22).

Different strategies can support and enhance all steps of this treatment process, which will be described in detail in this article. This review aims to give a comprehensive overview of *in situ* vaccines for the treatment of HCC in the context of the underlying immune dysfunction and immunosuppressive TME. Both preclinical and clinical *in situ* vaccine strategies and techniques will be discussed, highlighting opportunities as well as potential limitations and pitfalls of this immunotherapeutic approach.

### Liver and HCC Immunology

Many challenges in treating hepatic malignancies originate from the tolerogenic nature of hepatic immune responses and are aggravated by distinct immunosuppressive effects conferred by the tumor and its TME (23, 24). The liver is in continuous contact to non-self antigens from the portal tract and hepatic immune tolerance is the ordinary response to non-self structures, unless they are accompanied by distinct danger signals (25).

Antigen presentation in the liver can be performed by professional APCs such as DCs as well as liver-specific APCs, e.g. hepatic stellate cells, Kupffer cells, liver sinusoidal endothelial cells and even hepatocytes (26). Due to an overlap of markers, e.g. Kupffer cells and other macrophages in the mouse liver can express the “DC marker” CD11c or MHC-II molecules, it is particularly challenging to dissect the contribution of different myeloid APCs in the liver. This is even more difficult in diseased liver, as liver injury (or tumor development) commonly leads to a strong recruitment and accumulation of myeloid cells in the liver (27).

DCs, the most important professional APCs, usually have an immature phenotype in the liver. They can interact with T cells directly in the liver or migrate to the draining lymph node to present antigens there (28). While the exact significance of the place of antigen presentation – directly in the liver, especially in proximity to portal tracts, or after DC migration to lymph nodes – is not entirely clear in HCC, it has been shown that DC-mediated T cell activation can occur in both localizations (29, 30).

The main subsets of DCs include conventional (cDC) and plasmacytoid DCs (pDC). Type 1 conventional DCs (cDC1) are capable of cross-presenting extracellular antigens in a MHCI-restricted manner to CD8+ cells (31), a process that, depending on the state of DC maturity and concomitant expression of costimulatory molecules or tolerogenic signals can result either in T cell cross-tolerance or in an efficient T cell cross-priming with ensuing cytotoxic activity (32). The latter makes the cDC1-CD8+ T cell interaction essential for tumor recognition and the initiation of antitumor immune responses.

While mutated neoantigens are rarely presented on HCC cells (33), TAAs such as alpha-fetoprotein (AFP), glypican-3 (GPC-3) or New York esophageal squamous cell carcinoma-1 (NY-ESO-1) are oftentimes overexpressed in HCC and phagocytosed by APCs (34, 35). Nevertheless, DCs are often unable to effect successful T cell cross-priming, with multiple underlying mechanisms of dysfunction, including DC immaturity or “semi-maturity” (36), the induction of a tolerogenic DC phenotype by tumor-derived factors (37) and the expression of immune checkpoints (38, 39). These mechanisms culminate in DCs that either fail to activate specific T cell responses or even promote specific immune tolerance, leading to a suppression of CD8+ T cell responses and to cancer immunosurveillance failure (37).

**Figure 2:**
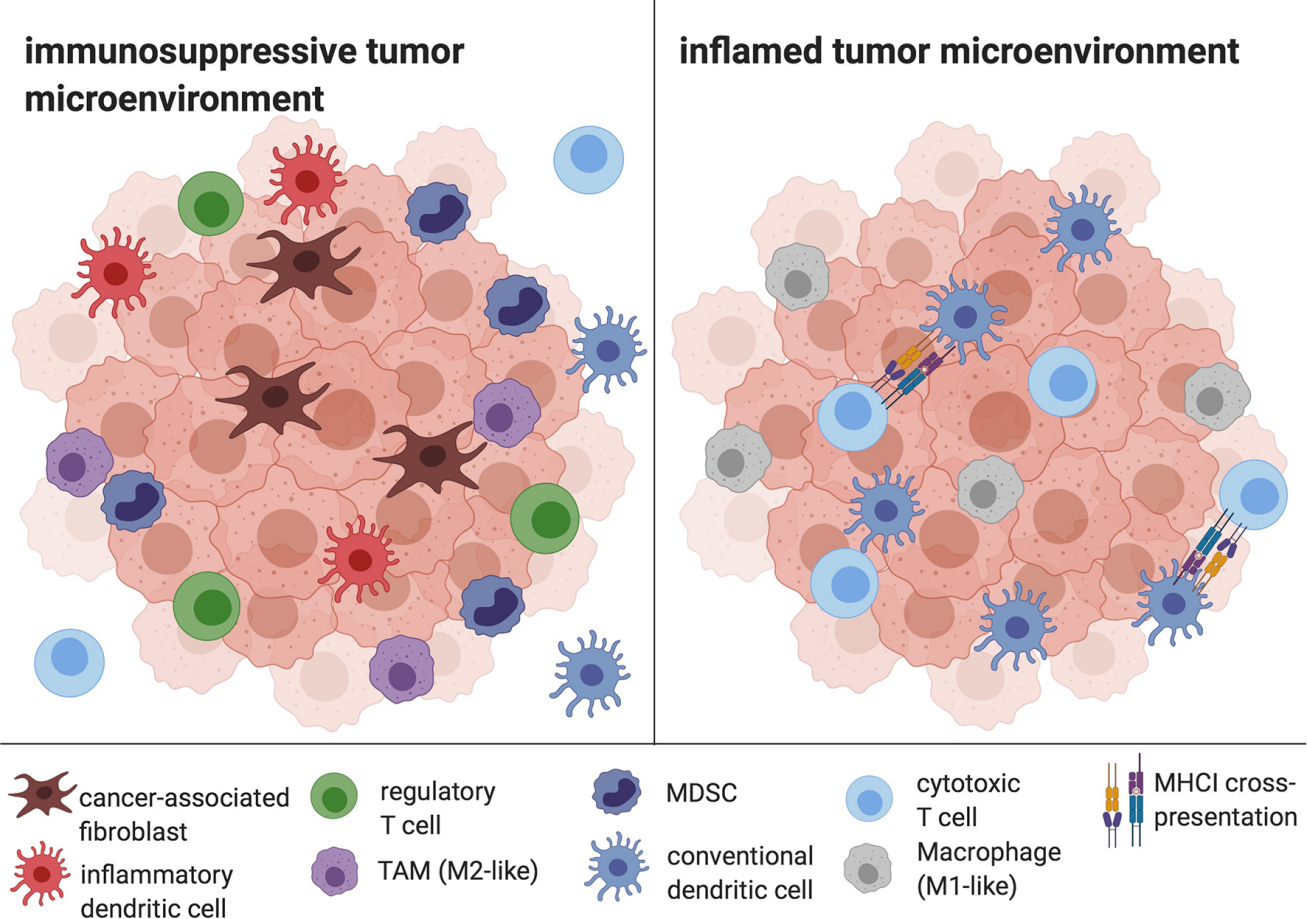
The HCC TME. Cancer-associated fibroblasts, infDCs, TAMs, Tregs and MDSC mediate immune evasion and prevent APC and CD8+ T cell infiltration and efficient antigen presentation. In contrast, an inflamed tumor microenvironment is characterized by the depletion of tolerogenic cells and the infiltration of DCs, CD8+ cells and M1-like macrophages, enabling antigen cross-presentation and cytotoxic activity. Created with biorender.

The TME of HCC is composed of immune cells such as tumor-associated macrophages (TAM), myeloid-derived suppressor cells (MDSC), regulatory T cells (Tregs), inflammatory DCs, as well as stromal cells like cancer-associated fibroblasts and significantly contributes to cancer immune evasion (40, 41). Typical effects include the disruption of essential DC functions like DC maturation, phagocytosis and migration as well as the inhibition of T cell responses (42), but also the promotion of angiogenesis and tumor growth (43). Furthermore, the TME supports Th17 responses, with a resulting aggravation of the underlying chronic liver inflammation on the one hand, and, on the other hand, with proangiogenic effects (44). The HCC TME is considered to be one of the central determinants of therapy resistance, for example against Sorafenib (45). The principle of overcoming the inhibitory effects of tumor cells and their TME by harnessing the DC-T cell axis has evolved into several promising therapeutic approaches, including *in situ* vaccines (**Figure 2**) (17).

### Inducing Immunogenic Cell Death

Immunogenic cell death (ICD) is a stress-induced, regulated type of cell death that triggers an adaptive immune response (46). It is characterized by the release of cellular antigens, which are taken up and presented by APCs and immune activation depends on sufficient antigenicity and adjuvanticity (47, 48). Antigenicity is determined by the quality and quantity of TAAs, while adjuvanticity is determined by the simultaneous release of danger signals such as damage-associated molecular patterns (DAMPs) (49). Immunostimulatory DAMPs include a release of endoplasmatic reticulum proteins like calreticulin and heat shock proteins (HSP), the toll-like receptor (TLR) 4 and TLR9 agonist high mobility group box 1 (HMGB1) and ATP, leading to DC recruitment and activation at the site of tumor cell death (50). For *in situ* vaccination, ICD provides an elegant method to harness the whole breadth of available cancer antigens for an immune response. In preclinical and clinical settings, various endogenous and exogenous stimuli can trigger ICD, including several conventional chemotherapeutic agents (51), radiation therapy (52) as well as therapeutic oncolytic viruses (53), which have already been described for *in situ* vaccination approaches and will be discussed in detail below. Nevertheless, triggers such as radiotherapy might also induce immunosuppressive changes in the TME (54) which has to be taken into account while developing *in situ* vaccination protocols for HCCs.

#### Oncolytic Viruses

Oncolytic viruses exhibit a tropism for malignant cells, selectively infecting tumor cells while sparing normal cells. They replicate inside and lyse cancerous cells, releasing TAAs in an immunogenic fashion with simultaneous release of DAMPs and pathogen associated molecular patterns (PAMPs) (53, 55). The concomitant expression of different transgenes can mediate additional immunomodulatory effects. For example, human granulocyte-macrophage colony-stimulating factor (GM-CSF) has been integrated into the viral genome to accompany viral replication with GM-CSF production to recruit APCs and promote their maturation (see also section on *Recruiting and Activating APCs*). Subsequent cross-priming of CD8+ T cells induces a cytotoxic response with ensuing systemic effects, and, ideally, accompanied by a memory response with long-lasting immunity (56). Various viral strains have been described as potential antitumor vaccines, each conferring different (side-) effects (21).

The first oncolytic virus to gain regulatory approval in the USA as well as in the European Union and Australia was talimogene laherparepvec (T-VEC), a modified herpes simplex virus (HSV) 1 expressing GM-CSF for intralesional injection of advanced malignant melanoma (57). A phase Ib/II trial combining intratumoral T-VEC with Pembrolizumab is currently investigating the injection of T-VEC into HCC and hepatic metastases (MASTERKEY-318, NCT02509507). Based on these advances, an HSV-1-based oncolytic vector (Ld0-GFP) was engineered to trigger ICD both *in vitro* and in mice models, where Ld0-GFP induced tumor eradication in over 60% of established hepatomas (58).

To date, the oncolytic pox virus vaccine JX-594 expressing the transgenes GM-CSF and β-galactosidase is the oncolytic virus with the most clinical evidence in HCC (see **Table 1**) (23). Observed effects of JX-594 application included a T cell response against vaccinia, β-galactosidase and TAAs such as MAGE-A1, MAGE-A3 and survivin in a subset of patients (53). Further, polyclonal antibody-mediated cytotoxicity was also suggested as a driver of antitumor activity (60). A disruption of tumor vasculature, mediated by a selective infection of tumor-associated vascular endothelial cells in murine tumors and human HCC, has been identified as an additional mechanism of action. As such, vaccinia exploits high cellular thymidine kinase levels to replicate, a process that is enhanced in tumor-associated vasculature *via* VEGF and other mediators (64). Encouraging clinical results were achieved with intralesional injections of JX-594 in 10 patients with advanced primary and metastatic liver tumors in a phase-I setting over a decade ago (59). A subsequent dose-finding study in subjects with advanced HCC suggested an improved OS for intravenous high-dose JX-594 application (60). However, a Phase IIb trial in sorafenib-experienced patients did not show a superior OS of the JX-594-group compared to best supportive care (53). Hoping that JX-594 therapy may induce a T-cell response that overcomes an immunosuppressive TME and increases sorafenib responsiveness, the PHOCUS phase III trial (NCT02562755) compared sorafenib treatment with vaccinia virus-based immunotherapy, followed by sorafenib. The results of an interim futility analysis, however, led to the termination of the study because it was considered unlikely that the trial would meet the primary endpoint, OS (65). A phase I/IIa trial combining JX-594 with Nivolumab as first-line treatment of advanced HCC is still ongoing (NCT03071094).

**Table 1:** Clinical Trials on *in situ* vaccines in HCC/solid tumors.

Number	Type of cancer	Phase	Substance	Name	Application	Combination Therapy	Patients	Status	Oncological Findings	Immunological Findings	Year	Ref.
*Oncolytic Virus*												
NCT02509507	HCC, liver metastases	I/IIb	oncolytic herpes virus expressing GM-CSF	Talimogene Laherparepvec (T-VEC)	IT	Pembrolizumab IV	206	recruiting			2015	
NCT00629759	HCC, liver metastases	I	oncolytic pox virus (thymidine kinase deleted vaccinia virus) + GM-CSF	Pexastimogene Devacirepvec (Pexa-Vec, JX-594)	IT		14	completed	30% partial response, 60% stable disease, 10% progressive disease (RECIST) 80% objective response by Choi criteria	induction of white blood cells and cytokine release (IL-6, IL-10, TNF-α) development of anti-JX-594 antibodies	2006	(59)
NCT00554372	HCC	IIa	oncolytic pox virus + GM-CSF	JX-594	IT		30	completed	15% objective response, 50% intrahepatic disease control rate (mRECIST) 62% Choi response rate OS significantly higher in high-dose compared to low-dose group	induction of antitumoral immunity (*in vitro* antibody-mediated complement-dependent cytotoxicity against HCC cell lines) induction of cytotoxic T cell activity to vaccinia peptides & JX-594 transgene product	2007	(60)
NCT01171651	HCC (Sorafenib naive)	II	oncolytic pox virus + GM-CSF	JX-594	IV/IT		25	completed	significant decrease of tumor perfusion in both injected and non-injected tumors	n.a.	2010	(61)
NCT01387555	HCC (PD under Sorafenib)	IIb	oncolytic pox virus + GM-CSF	JX-594	IV/IT	Best supportive care	129	completed	no improvement of OS, response rate, time to progression compared to best supportive care alone	T cell proliferation T cell response to vaccinia peptides & TAAs	2011	(53)
NCT02562755	HCC (Sorafenib naive)	III	oncolytic pox virus + GM-CSF	JX-594	IT	Sorafenib PO	459	completed			2015	
NCT03071094	HCC	I/IIa	oncolytic pox virus + GM-CSF	JX-594	IT	Nivolumab IV	30	active, not recruiting			2017	
NCT02293850	HCC	I	oncolytic adenovirus expressing hTERT promotor	Telomelysin (OBP-301)	IT		18	recruiting			2014	
*TLR agonists*												
NCT02556463	solid tumor	I	TLR 7/8 agonist	MEDI9197	IT	Durvalumab IV and/or radiotherapy	53	terminated	no objective clinical response (19 and 28% disease control rates)	increased intratumoral CD8+ & PD-L1+ cells induction of type 1 and 2 IFN & TH1 response increased TLR7/8 downregulated genes	2015	(62)
NCT02668770	solid tumor	I	TLR9 agonist	Levitolimod (MGN1703)	SC/IT	Ipilimumab IV	55	active, not recruiting			2016	
*Interleukins*												
NCT01417546	solid tumor	I	fusion protein of IL-12	NHS-IL12	SC		83	recruiting	6% partial response, 40% stable disease, 54% progressive disease (RECIST)	IgG isotype antibodies *in vitro* induction of antibody-dependent cellular cytoxicity	2011	(63)
NCT03946800	solid tumor	I	IL-12 mRNA	MEDI1191	IT	Durvalumab IV	87	recruiting			2019	
NCT02960594	solid tumor (high risk of relapse after curative therapy)	I	DNA-based vaccine encoding IL-12	INO-9012	IM	other DNA vaccines	93	completed			2016	

IM, intramuscular; IT, intratumoral; mRECIST, modified ‘Response Evaluation Criteria in Solid Tumors’; mRNA, messenger RNA; SC, subcutaneous.

Patients included in the studies had locally advanced/metastases not suitable for resection and had progressive disease under standard therapies or contraindications, if not otherwise indicated.

A different approach in oncolytic viruses harnesses the high telomerase activity of malignant tumors to selectively infect tumor cells. The oncolytic adenovirus variant Telomelysin successfully induced ICD, recruitment of CD8+ T cells and inhibition of intratumoral Foxp3+ lymphocyte infiltration. When combined with PD-L1 blockade, Telomelysin A caused systemic tumor regression in subcutaneous murine pancreatic and colon cancer models (66). Currently, a phase I study (NCT02293850) is recruiting patients with HCC to evaluate safety and efficacy of Telomelysin.

New virological engineering methods have yielded several novel, elegant concepts of oncolytic virus therapy (see **Table 2**). For example, engineering hybrid vectors has been proposed to circumvent distinct side effects of the individual viral strains. Thus, a recombinant virus from vesicular stomatitis virus (VSV) and Newcastle disease virus (NDV) (r-VSV-NDV) combined the rapid replication of VSV with the efficient ICD-induction of a fusogenic virus while avoiding the safety and environmental concerns associated with the parental vectors. Mice with orthotopic HCC tumors showed prolonged survival under r-VSV-NDV therapy, with an enhanced safety profile compared to the parental strains (67). Another recent development in oncolytic virus therapy not yet investigated in humans is the integration of programmable and modular synthetic gene circuits into an adenovirus vector. A hierarchical assembly method combines tumor lysis with a controlled expression of the immune effectors GM-CSF and interleukin (IL)-2, as well as single-chain variable fragments against the checkpoint inhibitors PD-1 or PD-L1. Both *in vitro* and *in vivo* xenograft models showed antitumor efficacy and HCC tumor regression. Mice treated with the synthetic oncolytic adenovirus were protected from HCC tumor rechallenge and had significantly increased intra-tumoral lymphocytes, as well as a significantly higher proportion of interferon (IFN)-γ producing and Ki67+ CD8+ T cells (74).

**Table 2:** *In vivo* studies on *in situ* vaccines in HCC.

Substance (Name)	Application	Tumor model	Findings			Ref.
			oncological	immunological		
*Oncolytic virus*						
HSV-1 based oncolytic vector (Ld0-GFP)	IT/IV	SC xenograft nude mice model (Huh7, Hep3B) syngeneic HCC mouse model orthotopic HCC mouse model (H22)	inhibited tumor growth/tumor size reduction	n.a.		(58)
VSV-NDV hybrid vector with glycoprotein exchange	IV	transgenic AST mice (liver-specific albumin promoter, loxP-flanked stop cassette, SV40 large T antigen oncogene) immune-deficient NOD-SCID mice	prolonged OS in tumor-bearing mice safe in immune-deficient mice	tumor-specific viral syncytium formation leads to tumor ICD		(67)
oncolytic adenovirus encoding TRAIL and IL-12	IV	orthotopic xenograft (Hep3B) in athymic nude mice	tumor regressions/necrosis	apoptosis promotion, activation of caspase-3 and -8 IFN-γ upregulation NK cell and APC infiltration VEGF and CD31 (tumor microvessel) repression		(68)
*Bacteria/bacterial products*						
Clostridium novyi-NT spores with iron oxide nanoclusters	Rats: IT Rabbits: *via* the hepatic artery	Rats: N1-S1 inoculation Rabbits: VX2 tumor (orthotopic inoculation)	spore delivery to tumor is feasible	oncolytic activity		(69)
*Chemotherapeutics*						
Icaritin + Doxorubicin + Lenvantinib	IV Lenvantinib orally	hemisplenic hepatoma (Hepa1-6) mouse model	synergistic inhibition of tumor growth protection against tumor rechallenge	upregulated CD8+ and CD4+ T cells, activated DC cells and memory T cells downregulated MDSC, Treg, and M2-like macrophages		(70)
*Flt3L*						
radio-inducible suicide gene therapy (+CD40-L/) + Flt3-L gene therapy	IP	orthotopic hepatoma (BNL transfected with radiation-inducible promoter-controlled HSV-TK) in mice	increased OS, inhibition of tumor growth and cure protection against tumor rechallenge		upregulated activated CD8+ T cells, upregulated CD4+ T cells and NK cells Th1 polarization	(71, 72)
defective adenovirus expressing Flt3L + 5FU	Adenovirus: IT 5FU: IP	SC hepatoma (Hepa1-6) in mice	tumor growth inhibition cure of established tumors tumor-specific immunity can be adoptively transferred between animals by transfusing CD3+CD8+ T cells		elevated intratumoral DCs NK cells and lymphocytes	(73)
*GM-CSF*						
Adenovirus with synthetic gene circuits with GM-CSF/checkpoint blockade expression	IT	xenograft nude mice model (Huh7, HepG2) s.c. hepatoma (Hepa1-6) model	inhibited tumor growth protection against tumor rechallenge		increased IFN-γ+ and Ki-67+ cells among the tumor infiltrating CD8+ T cells	(74)
Adenovirus encoding GM-CSF/IL-12	hepatoma: IT DEN-induced tumors: *via* hepatic artery	Mouse: orthotopic hepatoma (BNL) Rat DEN model	Synergistic tumor regression		CD8+ T cells, NKT cells, and macrophages exert antitumor functions, elevated IFN-γ	(75)
*TLR agonists*						
TLR9 agonist + anti-PD-1/ anti-PD-L1	IP	SC and orthotopic hepatoma (Hepa1-6) in mice	Synergistic inhibition of tumor growth		TLR9 signaling promotes PD-L1 transcription	(76)
HMGN1 + TLR7/8 agonist (R848/resiquimod) + Anti-CTLA4/anti-PD-L1/Cytoxan	HMGN1, R848, Anti-CTLA4, anti-PD-L1: IT Cytoxan: IP	SC hepatoma (Hepa1-6)	cured hepatomas, protection from tumor rechallenge		tumor-specific CD8+ T cells, elevated CXCL9, CXCL10, and IFN-γ expression in the tumor, tumor T cell infiltration	(77)
*Interleukins*						
Lipid nanoparticles delivering IL-12 mRNA	IV	*LAP*-*tTA/tet-O-hMYC* transgenic mice (MYC-driven HCC)	reduced tumor burden and prolonged OS		increased splenic volume and inducted IFNγ mRNA recruitment of CD44+ CD3+ CD4+ Th cells	(78)
radiation + adenoviral vector encoding IL-12	IT	SC or orthotopic hepatoma (BNL, BNL-P2)	tumor regressions and systemic effects against distant tumors		upregulated MHC class II, CD40 and CD86 on tumor-infiltrating DCs; Reduction of MDSCs and ROS Activated intratumoral CD8+ T and NK cells	(79)
*Checkpoint Inhibitors*						
Radiation + anti-PD-L1	Injection (not specified)	IM inoculation (HCa-1)	combination treatment significantly suppressed tumor growth, significantly improved OS		radiation upregulated tumor PD-L1 expression increasing apoptosis, decreasing tumor cell proliferation, restoration of CD8+ T cell functions	(80)

IM, intramuscular; IP, intraperitoneally; IT, intratumoral; IV, intravenous; mRNA, messenger RNA; SC, subcutaneous.

Experimental animals are wild type mice, if not otherwise indicated.

#### HMGB1 and HMGN1

The nonhistone chromatin-binding proteins HMGB1 and high-mobility group nucleosome binding domain 1 (HMGN1) are involved in the regulation of cell death and survival. In the extracellular milieu, HMGB1 and HMGN1 function as alarmins that contribute to the immunogenicity of cell death. HMGB1 is released from damaged cells due to the permeabilization of nuclear and plasma membranes and binds to receptors on immune cells such as TLR2, TLR4 and receptor for advanced glycation endproducts (RAGE) (49), while HMGN1 predominantly binds to TLR4. Both HMGB1 and HMGN1 convey pro-inflammatory effects including DC activation, Th1 polarization and the enhancement of T cell antitumor responses (81, 82).

The prominent role of HMGB1 in this context was illustrated in murine anti-tumor vaccination models, where HMGB1 blockade abrogated therapeutic effects both *in vivo* and *in vitro* (83, 84). Because of its ability to activate DCs, synthetic HMGB1 peptides have been investigated as adjuvants to enhance the immunogenicity of vaccines, both against infectious agents and tumors (85–87). Concerns about the intratumoral application of HMGB1 in malignant tumors are based on the observation that reactive oxygen species, which are often elevated in the TME, can oxidize HMGB1 and neutralize its immunostimulatory activity (88). Furthermore, the immune checkpoint receptor T-cell immunoglobulin and mucin-domain containing-3 (TIM-3) on tumor-associated DCs was able to abrogate therapy-induced immunogenicity of cell death by interacting with HMGB1. The inhibition of uptake of nucleic acids from dying, chemotherapy-treated tumor cells into DC endosomes resulted in lower immunogenicity (89). Of note, HMGB1 expression is elevated in tumors and serum of HCC patients and its expression inversely correlates with survival (90, 91). A contribution of HMGB1 and its receptor(s) to HCC carcinogenesis has been suggested by several sources (92, 93) and *in vitro* data showed that HMGB1 enhanced the ability of proliferation, migration and invasion of HCC cells (94). So far, to our knowledge, HMGB1 has not been explored as an adjuvant for *in situ* vaccines for HCC, probably owing to its Janus face in tumorigenesis, TME immunosuppression and DC-T cell crosstalk.

In 2012, Yang et al. first reported that extracellular HMGN1 significantly contributes to T cell antitumor immunity, with a central role in antigen-specific immune responses (95). Since then, HMGN1 has been successfully explored as an HCC vaccine adjuvant, both in *ex vivo* settings (96) and in *in situ* concepts. Thus, the intratumoral delivery of HMGN1, the TLR7/8 agonist Resiquimod and checkpoint inhibitors cured established subcutaneous hepatomas and protected the mice against tumor rechallenge (see also *TLR7/8*) (77).

#### Bacteria and Their Products

While the dysregulation of the gut microbiota in HCC and chronic liver disease has received significant attention (97), few authors have explored bacterial immunotherapy for HCC. The most established bacterial immunotherapy for solid tumors is Bacillus Calmette-Guerin (BCG). It is routinely used intravesically in bladder cancer as an immunogenic adjuvant treatment after resection of high-grade, early-stage tumors (98, 99), while the search for targeted treatments for these tumors are still ongoing (100, 101). BCG induces multifaceted immunological effects. Multiple BCG component agonists mediate an innate response by activating TLR2, 4 and Dectin-1 and 2, host sensors on diverse immune cells including CD14+ monocytes and neutrophils (102). Pattern recognition receptors (PRR) on APCs are activated by BCG, leading to TLR activation and antigen presentation with CD4+ and CD8+ cell activation (102). Furthermore, BCG confers a direct cytotoxic effect on cancer cells, inducing oxidative stress and resulting in ICD, reflected in the release of HMGB1 (103). The possibility of localized intravesical administration has corroborated its role in bladder cancer, but BCG has also been investigated for the treatment of other cancer entities, with data in HCC limited to case reports, such as the successful therapy combination of BCG, IL-2 and melatonin (104).

Based on the rationale that gram-positive bacteria activate DCs *via* TLR2 signaling and that anaerobic bacteria could thrive in the hypoxic TME, bacteriolytic therapy with *Clostridium* species has been suggested as a potential inductor of tumor ICD (105). While intravenous administration causes severe side effects, rat and rabbit models confirmed that both intratumoral injection and intra-arterial transcatheter infusion of *C. novyi* into hepatic malignancies are feasible (69, 105). *In vitro* assays showed that *C.novyi*-treatment resulted in oncolysis and a significantly decreased metabolic activity of rodent HCC cell lines (69).

#### Radiotherapy

Radiotherapy directly induces DNA damage and is a widely used cancer treatment in both curative and palliative settings (106). While whole liver toxicity limits the application of external-beam radiotherapy for HCC treatment (107, 108), selective approaches like stereotactic body radiation therapy, radioembolization and selective internal radiation therapy constitute valid local clinical treatment options for HCC, but their efficacy is limited by extrahepatic spread and tumor manifestation outside the irradiated field (6, 109). While abscopal effects are limited to case reports in HCC (110, 111), a growing body of evidence points towards the induction of ICD and a modulation of the TME through radiotherapy (54, 106). Thus, the effects of radiotherapy have not only been linked to DNA damage, but also to TAA release, DAMP secretion, TLR4 activation on DCs and ensuing cross-presentation to CD8+ T cells (83, 112). Furthermore, an upregulation of the chemotactic C-C chemokine ligand (CCL)5 and CXC-ligand (CXCL)16 pathway and an increased infiltration of CD8+ T cells and Natural Killer (NK) cells into the tumor were observed in HCC patients undergoing Yttrium-90 radioembolization, along with an increase of APCs and CD4+ and CD8+ cells in peripheral blood (113). However, radiotherapy also confers subsequent dosage- and fractionation-dependent immunosuppressive effects on the TME (54). This includes recruitment of Tregs to the TME and a “M2-like polarization” of TAMs (54), as well as an increased tumor PD-L1 expression and a heightened fraction of exhausted PD-1+/TIM-3+ CD8+ T cells (80, 113). In this regard, the combination with systemic immunomodulators and checkpoint inhibitors is a pervasive strategy to re-establish immunosurveillance (54) that so far has only been explored preclinically (80) and in small nonrandomized settings with encouraging results (114, 115). Murine colon carcinoma tumor models showed that low-dose radiotherapy-mediated tumor PD-L1 expression is induced by CD8+ T cell IFNγ signaling and peaks at 72 hours after treatment. Here, combination treatment with checkpoint inhibitors (anti-PD-1 or anti-PD-L1, respectively) was most effective when administered concomitantly (116). Several *in situ* vaccine regimens have harnessed radiotherapy as an inducer of ICD in HCC, including promising combinations of radiotherapy with IL-12 (see also section on *Optimizing Cross-Presentation and T Cell Priming*) (79).

#### Chemotherapy and Transarterial Chemoembolization (TACE)

Several chemotherapeutic agents are more effective in immunocompetent hosts because they induce ICD and favorably modulate the TME (117, 118). While many pathway inhibitors and chemotherapy regimens do not confer a survival benefit in HCC and negatively impact liver function in chronic liver disease while conveying considerable side effects (107, 119–122), exploring chemotherapeutic agents as triggers of ICD may require dosage adaption and addition of immunomodulators (123). Therefore, chemotherapeutics without a positive clinical effect in conventional HCC therapy may still be implemented for *in situ* vaccine concepts to, firstly, trigger ICD and, secondly, to modulate the TME. Furthermore, the local application of chemotherapy in combination with embolizing agents – transarterial chemoembolization (TACE) – has emerged as a selective and valid treatment option (124).

Several sources have confirmed that chemotherapy agents can induce ICD in cancer cells. *In vitro* experiments showed that anthracyclines promote ICD in tumor cells by inducing the translocation of calreticulin, HSP70 and 90 to the cell surface and promoting HMGB1 release (125). Their stimulation of TLR3 results in a rapid type I IFN production, with subsequent CXCL10 release (126). Doxorubicin, widely implemented in TACE, induced ICD in HCC cell lines, however, with a weak effect on immune cells (70, 127). This effect was augmented by adding the mitophagy-inducing drug icatirin, which resulted in protection from tumor rechallenge. Synergistic effects of icatirin and doxorubicin furthermore included a remodeling of the TME, with an upregulation of CD8+ and CD4+ T cells, memory T cells and activated DCs, while the numbers of MDSCs, Tregs, and M2-polarized macrophages decreased (70). The cytokine profile showed decreased levels of CCL2, TGFβ, IL-4, IL-6, IL-10 and increased levels of IFNγ, tumor necrosis factor (TNF)-α, and IL-12, with the latter a potent inducer of a T helper (Th)-1 phenotype (70, 128). Similarly, oxaliplatin, also clinically used for TACE, has been shown to promote ICD *in vitro* and to induce DC maturation as well as increase CD8+ T cells in an HCC inoculation mouse model (129). A recent study has linked therapeutic resistance to oxaliplatin-based TACE to the density of infiltrating TAMs, since HCC cells co-cultured with macrophages showed higher oxaliplatin-resistance *in vitro*. Furthermore, HuH7 xenografts co-implantated with THP-1 derived macrophages responded significantly less to oxaliplatin treatment in a murine tumor model (130).

Modulating the TME can contribute to the success of *in situ* vaccination of solid tumors, and several chemotherapeutic agents are able to restore an efficient antitumor response by depleting or changing immunosuppressive cell populations. An early report showed that low-dose cyclophosphamide selectively depleted CD4+CD25+ Tregs, restoring peripheral T cell proliferation and NK cell killing activities (131). Additionally, cyclophosphamide-induced ICD expanded the cDC1 compartment and facilitated cross-priming of T cells (132). At the same time, cyclophosphamide was also reported to expand CD11b+ Ly6C^hi^ CCR2^hi^ MDSCs that inhibited long-term tumor control in a murine lymphoma model through the PD-1-PD-L1 axis (133).

Depletion of MDSCs has been attributed to several chemotherapeutics, including doxorubicin (134), cisplatin (135) and oxaliplatin (136). Oxaliplatin treatment also increased intratumoral T-cell infiltration (including Tregs) in mice (137), while other studies suggested that platinum-based therapies promote TAMs by enhancing M2 polarization (138).

A serious immunological concern regarding systemic chemotherapy is systemic immune suppression because of myelo-and lymphopenia, especially when dosage approaches the maximum tolerated dose (139). However, in clinical reality, routine regimens usually employ significantly lower doses and do not impair systemic vaccination immune responses, as demonstrated by Wumkes et al. in cohorts of chemotherapy-treated patients with solid tumors who had adequate responses to influenza vaccination (140).

Several chemotherapeutic agents have already been harnessed to improve the efficacy of immune checkpoint inhibitors in other tumor entities. Cisplatin was able to sensitize triple negative breast cancer to PD-1 blockade (141), while 5-fluorouracil plus oxaliplatin (FOLFOX) combined with checkpoint blockade showed strong synergistic effects, because FOLFOX induced PD-1+ cytotoxic T cell infiltration (142). In a syngeneic HCC mouse model, the combination of oxaliplatin and anti-PD-1 antibodies inhibited tumor growth better than the respective monotherapies (129).

Probably due to the minor role of systemic chemotherapy in HCC treatment, only few studies have explored chemotherapy within *in situ* vaccination models. Intratumoral application of an adenovirus expressing Fms-like tyrosine kinase 3 ligand (Flt3L) together with 5-fluorouracil in a murine hepatoma model induced complete remission of established tumors (see **Table 2**) (73).

#### Sorafenib

Besides exerting anti-proliferative and anti-angiogenic effects by inhibiting VEGFR, PDGFR and RAF (143), the multikinase inhibitor sorafenib can also induce autophagy-mediated ICD. As such, sorafenib mediates ferroptosis, a regulated form of ICD that results from a decreased antioxidant capacity, coupled with iron overload and massive lipid peroxidation (144). Sorafenib-induced ferroptosis was shown to be accompanied by a HMGB1 release with subsequent inflammation (145), underlining the potential of Sorafenib-induced cell death in *in situ* vaccine concepts.

At the same time, dose-dependent effects of Sorafenib on antitumor immunity have been noted, with high-dose Sorafenib reported to increase the proportion of PD-1 expressing CD8+ T cells and resulting in less intratumoral T cell infiltration in a woodchuck hepatitis virus-induced HCC model (146). In vitro, subclinical Sorafenib doses selectively increased CD4+ CD25- effector T cell activation and blocked Treg function in PBMCs from HCC patients (147). This concept has been applied to a murine adoptive T cell therapy, where low-dose Sorafenib both enhanced function and migration of transferred CD8+ T cells and decreased the number of MDSCs and Tregs in the TME (148).

### Recruiting and Activating APCs

#### Flt3L

Flt3 is essential to the regulation of homeostatic DC development in the bone marrow and lymphoid organs and the upkeep of sufficient numbers of peripheral DCs (149–151). Administration of recombinant Flt3L leads to an additional mobilization from the macrophage DC progenitor compartment (149), an effect that has been confirmed in both healthy volunteers and cancer patients (152–154). Furthermore, Flt3L injection combined with polyinosinic:polycytidylic acid (polyIC), a TLR3 agonist, induced the expansion and activation of CD103+ DC progenitors (cDC1) in a murine melanoma model, leading to an increased sensitivity to checkpoint blockade (155).

Oncolytic viruses expressing Flt3L have been investigated in an animal model of *in situ* vaccination (71). Kawashita et al. demonstrated that radio-inducible suicide gene therapy, using a cytotoxic expression vector of herpes simplex virus thymidine kinase controlled by a radiation-inducible promoter, was significantly enhanced in its efficacy by addition of a recombinant adenovirus expressing human Flt3 ligand (Adeno-Flt3L) in a hepatoma mouse tumor model. Adeno-Flt3L led to a Th1-polarized immune response with activation of cytotoxic CD8+ T cells. Additional boosting of the antitumor response was achieved with the addition of Adeno-CD40L to enhance DC maturation, with mice that had cleared the tumor being protected from subsequent tumor rechallenge (71). Clinically, Flt3L- based *in situ* vaccines have been investigated in several malignancies, such as colon carcinoma and indolent non-Hodgkin lymphoma (iNHL) (84, 156). Thus, an *in situ* vaccine regimen consisting of Flt3L, radiotherapy and a TLR3 agonist induced systemic CD8+ T cell antitumor responses in a mouse model of iNHL and renewed the susceptibility to checkpoint blockade. Furthermore, a clinical trial exploring this *in situ* vaccination regimen (NCT01976585) reported durable clinical remissions in patients with iNHL. Immunological effects of this combination included the induction of TAA-laden, cross-presenting DCs and tumor infiltration of activated CD8+ T cells with upregulated PD-1 expression, which were responsive to anti-PD1 targeting (84).

Flt3L application dramatically expands cDC and pDC populations in peripheral lymphoid organs such as the liver. In a mouse model, Flt3L-induced DC expansion enhanced fibrosis regression in a matrix metalloproteinase (MMP)-9-dependent manner, implying its potential benefits even in cases of chronic injury and fibrotic remodeling (157). Therefore, Flt3L for HCC therapy may offer the opportunity to harness intrinsically elevated DAMPs to then induce the maturation of the recruited DC populations. Potentially, this may abrogate the need for DC-directed adjuvants, warranting the exploration of Flt3L in HCC.

#### GM-CSF

GM-CSF is a cytokine driving the differentiation, proliferation and activation of macrophages and DCs, with a polarization towards cDC1 and Th1 responses (158). The intra-tumoral application of GM-CSF has been validated in several solid tumors as a technique to attract and stimulate DCs. The systemic application is associated with considerable toxicities, and several trials have demonstrated the feasibility of intralesional injection in solid tumors such as malignant melanoma (159). A large trial in over 800 patients with resected malignant melanoma reported that GM-CSF monotherapy failed to confer clinical benefits in the adjuvant setting and did not enhance the response to an antitumor vaccine (160). Accordingly, the intralesional application of GM-CSF encoding agents has gained increased interest. The oncolytic pox virus vaccine JX-594 with the transgenes GM-CSF has been investigated for HCC with heterogeneous results (discussed in 3.1) (60). Another concept is the intra-tumoral injection of combination treatments with a GM-CSF and IL-12 encoding adenovirus. Here, GM-CSF monotherapy did not show significant therapeutic effects but was able to augment the efficacy of the IL-12 agonist. While IL-12 monotherapy only induced antitumoral NK cells, the addition of intratumoral GM-CSF succeeded in recruiting activated CD8+ T cells, NKT cells, and macrophages and achieved a higher rate of tumor regressions (see also *IL-12*) (75).

GM-CSF has also been implicated in HCC carcinogenesis, with an immunosuppressive effect on the TME. Accordingly, HCC patients presented with elevated GM-CSF levels in comparison to healthy controls (161). Ilkovitch et al. showed that GM-CSF injection in healthy adults leads to an expansion of MDSCs in the liver, effecting a heightened PD-L1 expression on Kupffer cells and an impaired IFN-γ production by activated T cells (162). In mice orthotopically implanted with Hepa1-6 cells, GM-CSF expression by tumor cells led to an infiltration with MDSCs, while neutralization of GM-CSF and IL-6 abrogated HCC progression in this model, with decreased MDSC and TAM infiltration (161). Though the effect of GM-CSF may be dependent on its spatiotemporal distribution in the TME, the observed effects may pose a potential pitfall of GM-CSF application in vaccine concepts.

#### Alarmins for DC Recruitment and Activation

Adjuvants to enhance DC immunogenicity hold promise to attract DCs to the tumor, augment antigen presentation, and polarize the ensuing response towards Th1 and cytotoxic T cells. A major group of agents harnessed to this aim are alarmins – endogenous intercellular signals that activate defense mechanisms and provoke an immune response via, amongst others, chemokine receptors (CCR) or TLRs (163). Besides their manifold influences on the innate immune response, some alarmins confer distinct effects on DC recruitment and maturation. As a consequence, DCs mature, upregulating CCR7, a process that facilitates their interaction with CCL19 and CCL21 and thus enables them to home to local lymph nodes (164). Some of the following chemotactic mediators and alarmins have been used individually, while others are integrated in multimodal *in situ* vaccination concepts.

When examining alarmins in the context of HCC and chronic liver disease, it should be noted that many of these pathways are severely dysregulated in this setting. Along other mechanisms of chronic inflammation, an increased gut permeability with translocation of intestinal bacterial components (PAMPs) typically causes a chronic TLR4-mediated inflammatory response and contributes to hepatocarcinogenesis (165). As several immunostimulatory agents proposed as adjuvants for *in situ* vaccines overlap with the preexisting chronic liver inflammation and with tumor-promoting pathways, a careful examination of these pathways is warranted in the context of HCC.

##### TLR3

Agonists of the TLR3 receptor include double-stranded RNA and single-stranded viral RNA with incomplete stem structures (166). TLR3 is highly expressed in the endosomal compartment of cDC1, and its stimulation induces cytokine and chemokine production, DC activation and maturation *via* the TLR3/TICAM-1 pathway and antigen cross-presentation (167, 168).

Modulation of the TME has been described as a potential effect of TLR3 signaling. Injection of polyIC, a dsRNA analog, resulted in a change of macrophage populations, converting “tumor-supporting macrophages” to “tumor suppressors”. The latter were characterized by M1-like polarization, TNF-α production and tumoricidal properties (169). However, polyIC is a ligand for multiple other PRRs besides TLR3, including protein kinase R, retinoic acid-inducible gene-I (RIG-I) and melanoma differentiation-associated gene 5 (MDA5), leading to severe systemic side effects (168, 170). TLR3 stimulation with attenuated systemic cytokine production was achieved using various other substances, including synthetic dsRNA derivatives (170) or dsRNA coupled to nanoparticle-based delivery systems (171). The former led to a Th1 polarization, reflected in elevated IL-12 production and CD8+ T-cell priming (172).

TLR3 receptor expression has been associated with improved patient survival in HCC and linked to chemokine-mediated intratumoral lymphocyte infiltration (173). In this line, loss-of-function polymorphisms of TLR3 were highly prevalent in HCC-bearing populations in comparison to controls (174). Moreover, a recent study by Bonnin and Fares et al. found that downregulation of TLR3 mediates resistance to apoptosis in HCC cells and is a potent escape mechanism. Interestingly, transgenic mice with an absence of TLR3 expression exhibited accelerated hepatocarcinogenesis without an altered tumor immune infiltrate (175).

While, to the best of our knowledge, no clinical trial currently investigates TLR3 agonists for HCC therapy, preliminary data from the NCT01976585 trial, an *in situ* vaccine approach including polyICLC (HiltonolTM) combined with checkpoint blockade in patients with indolent non-Hodgkin lymphomas showed encouraging response rates (84). Ongoing trials investigate the application of TLR3 agonists in other malignancies, among others, in advanced colorectal cancer in combination with pembrolizumab (NCT04119830), in malignant melanoma (NCT04093323) and in the neoadjuvant setting in malignant pleural mesothelioma (NCT04345705) (176).

##### TLR4

TLR4 is a receptor with a wide range of activating agents, including HMGB1, LPS, HSP60 and 70, that confers a broad variety of effects (177). While systemic LPS administration causes severe side effects, intra-tumoral applications have been suggested previously (178). Several studies reported that TLR4 agonists have been successfully harnessed as adjuvants in several models of other tumor entities like malignant melanoma and clinically, in BCG immunotherapy (179, 180). While *in vitro* activation of the surface TLRs 1/2 and 4 and the endosomal TLRs 3 and 9 has a similar activating effect on splenic DCs, *in vivo* data showed that stimulation of the surface TLRs 1/2 and 4 suppressed CD8+ T cell responses (181). Furthermore, TLR2 and TLR4 signaling increased the fraction of CD11c+ cDC2, which were defective in priming CD8+ T cells, and elevated IL-10 secretion and PD-L1 and PD-L2 expression on DCs (181, 182). An appealing explanation for this observation is that because endosomal TLRs are activated in viral infection, they promote cross-presentation, while this mechanism is not necessary in most bacterial infections, sensed by the surface TLRs 1/2 and 4 (181).

Similarly, LPS stimulation in the liver activated cDC2, the most prevalent DC subset in the liver, with ensuing IL10 secretion and almost no increase of proinflammatory cytokines. As a result, an increased production of Tregs from naive CD4+ cells and a promotion of a Th2 responses was reported (183). Tregs were also recruited *via* CXCL10/CXCR3 and TLR4 signaling in a rodent liver transplantation model, promoting HCC recurrence after ischemia-reperfusion injury (184). Furthermore, TLR4 signaling has been linked to HCC invasion, multidrug resistance, tumor angiogenesis and metastases, and TLR4 antagonists suggested as therapeutic modalities for HCC (185–187). To our knowledge, the role of *in situ* vaccine concepts with TLR4 agonists has not yet been clinically explored in HCC (188).

##### TLR9

Endosomal CpG motifs are recognized by TLR9, and the receptor can be targeted with nucleotides or nucleotide derivatives (188, 189). As a result, antigen-presenting cells are activated and CD8+ T cells differentiate into a terminal state of CD127lowKLRG1high effector cells with initial antitumor efficacy, but a limited lifespan (190). The latter observation may partially explain initially promising, but short-lasting clinical antitumor effects of TLR9 agonists (190).

A downregulation of TLR9 due to the single nucleotide polymorphism of the TLR9 promoter -1486T/C has been previously implied in impaired innate immunity (191), and also recently been associated with an increased risk of HCC recurrence after liver transplantation (192). At the same time, activated TLR9 signaling in tumor cells not only falls short of inducing an antitumor immune response, but even facilitates HCC survival. A synergy of HMGB1 and TLR9 was shown to up-regulate mitochondrial biogenesis of HCC cell lines and in murine HCC models under hypoxic conditions, promoting tumor survival and proliferation (193).

Several clinical trials investigating TLR9-agonist therapy reported negative results in small-cell lung cancer and in metastatic head and neck squamous cell carcinoma (194, 195). Subsequent murine studies showed additive treatment effects of a TLR9 agonist in combination with anti-PD-1 or anti-PD-L1 therapy in hepatoma cell lines and HCC (see **Table 2**) (76, 190). Of note, TLR9 agonism enhanced PD-L1 expression *via* PARP1 and STAT3, facilitating immune escape in the absence of checkpoint inhibition, but leading to synergistic effects in combination treatment (76). Moreover, in murine HCC models of anti-PD-1 nonresponders, TLR9 agonists were able to achieve durable remissions with systemic antitumor effects. CD8+ T cell proliferation with the generation of CD127highKLRG1low long-lived memory precursors and infiltration and the presence of IFN-γ and TNF-α signaling were observed after the combination of TLR9 agonist and checkpoint inhibition (190). Clinical studies of checkpoint inhibition combined with TLR9 agonists are underway for other cancer entities like malignant melanoma and B cell lymphoma (NCT02668770, NCT02254772).

A virus-like particle encapsulating a CpG-A TLR9 agonist (CMP-001) has recently been reported to cause tumor regression in syngeneic hepa1–6 mouse models of HCC, with a greater antitumor activity of CMP-001 monotherapy than that of sorafenib or PD-L1 blockade (196). While, to our knowledge, no clinical study is currently accruing patients for CMP-001 treatment in HCC, encouraging clinical data has been recently reported in malignant melanoma. As such, CMP-001 reversed PD-1 blockade resistance patients with progressive disease, resulting in an overall response rate of 23.5% (NCT02680184) (197), while the treatment combination of CMP-001 and Nivolumab (anti-PD-1) yielded an encouraging pathological response rate of 70% in the neoadjuvant setting in advanced melanoma (NCT03618641). This study observed an increased intra-tumoral infiltration of CD8+ T cells and CD303+ pDCs as well as elevated numbers of circulating activated PD1+/Ki67+ CD8+ T cells in patients with favorable response (198).

##### TLR7/8

The small molecules Imiquimod (TLR7 agonist) and resiquimod (TLR7/8 agonist) are widely recognized topical drugs applied for benign and malignant epithelial tumors (199) and cutaneous hematological malignancies (200). TLR7/8 stimulation results in an expansion of effector T cells, as well as an activation of DCs and NK cells (200). In preclinical HCC models, TLR7/TLR8 stimulation led to the maturation of DCs and to the promotion of IFNI/IL12-mediated activation of NK cells. Thus, the cytolytic activity of NK cells against HCC cells was significantly augmented *in vitro* and in HepG2 xenograft-bearing nude mice in the presence of monocyte-derived DCs (201). In a murine Hepa1-6 hepatoma model, a regimen consisting of HMGN1, resiquimod and a checkpoint inhibitor resulted in the elimination of established tumors and protected the mice against tumor rechallenge. The authors noted increased Hepa1-6-specific cytotoxic CD8+ T cells, CXCL9, CXCL10, and IFN-γ upregulation as well as an increased tumoral infiltration of T cells (77).

A recently published study investigated the combination of the TLR7 and 8 agonist MEDI9197 with PD-L1 inhibition with or without radiation therapy in various solid tumors, including one patient with HCC. While this regimen resulted in systemic and intratumoral immune activation with a Th1 and type 1 IFN gene expression signatures, intratumoral CD8+ T cell infiltration and tumor PD-L1 expression, none of the 52 included patients showed an objective response to treatment. Furthermore, while the use for superficial lesions was feasible, adverse effects were frequent when MEDI9197 was injected in visceral or deep-seated lesions, including death from hemorrhagic shock after injection into a liver metastasis (62).

### Optimizing Cross-Presentation and T Cell Priming

#### HSPs

HSPs are a family of proteins classified by molecular weight that chaperone the folding and translocation of proteins under cellular stressors such as infection, inflammation, toxins and hypoxia (202). The signaling effects of HSPs are highly dependent on its localization and binding partners. While high levels of intracellular membrane-associated Hsp70 in cancer cells are anti-apoptotic, extracellular soluble Hsp70 can trigger innate and adaptive immune responses. The ability of HSP to chaperone TAAs and facilitate their uptake by APCs with subsequently endorsed cross-presentation is central to their immunogenic effects. Furthermore, HSPs recruit leukocytes, polarize Th cell responses towards Th1 cells, activate NK cells as well as induce the maturation of DCs (163, 203). While reliable evidence that tumor-derived HSP-peptide complexes are able to enhance cross-presentation of TAAs has been brought forward by several studies, the exploration of their immunogenic effects may be warranted to boost *in situ* vaccination strategies.

#### IL-12

IL-12 is a potent regulator of adaptive T cell responses that activates cytotoxic T and NK cells, downregulates Th2 responses and induces a polarization towards Th1 responses (128, 204). Furthermore, IL-12 modulated the TME by converting monocytes into tumoricidal “M1-like” macrophages that inhibit HCC growth *in vitro* and in xenograft mouse models (205). While elevated IL-12 levels in HCC patients were associated with favorable clinical outcomes, the systemic application of IL-12 incurred dose-limiting toxicities, directing research efforts towards more sophisticated IL-12 delivery systems (78, 206). Delivering IL-12 *via* a messenger RNA (mRNA) lipid nanoparticle resulted in a reduced tumor burden in MYC-oncogene driven murine HCC. An increased infiltration of activated CD44+CD3+CD4+ Th cells into the tumor and an increased IFNγ production were observed in this model (78). An oncolytic adenovirus encoding human tumor necrosis factor-related apoptosis-inducing ligand (TRAIL) and IL-12 genes showed antitumor efficacy *in vitro* and in a murine xenograft model, with ensuing IFN-γ production and infiltration of NK cells and APCs. Furthermore, the combination led to a remodeling of the tumor microvasculature, with a repressed VEGF production, a decreased CD31 expression and reduced microvessel density (68). The adenovirus-mediated gene transfer of IL‐12 and GM‐CSF showed synergistic effects in orthotopic murine liver tumors and chemically induced multifocal liver tumors. Tumor regressions and a boost of IFN‐γ signaling, as well as an enrichment for CD8+ T cells, NKT cells and macrophages in the TME was reported (75).

Several clinical studies are currently investigating IL-12 therapy for solid tumors, including the application of an anti-DNA antibody-based fusion protein of IL-12 (NCT01417546), mRNA encoding for IL-12 and checkpoint blockade (NCT03946800), as well as an IL-12 DNA therapy combined with hTERT (NCT02960594) (see **Table 1**).

#### IL-2

Over 20 years ago, the systemic application of IL-2 was reported to achieve treatment responses in patients with metastatic renal cell carcinoma and malignant melanoma (207, 208). Since then, IL-2 has gained considerable attention for its potential to recruit and activate cytotoxic CD8 T cells and NK cells, to cause T cell proliferation and to induce polarization of the TME towards a Th1response. At the same time IL-2 activates and stimulates the proliferation of immunosuppressive Tregs *via* their CD25 receptor (209). Recently, an engineered IL-2 variant with abolished CD25 binding was reported to keep up its effects on CD8 T cells and NK cells, while evading the stimulatory impact on Tregs (210).

Several studies have suggested a protective effect of IL-2 against HCC development and recurrence. The high expression level genotype +114 TT was associated with a lower risk of HCC development in a hepatitis B positive cohort, while high peritumoral IL-2 levels were associated with a lower risk of tumor recurrence (211, 212). An ultra-low dose regimen of systemic IL-2 showed a moderate treatment efficacy in patients with advanced HCC, with an overall response rate of 16% (213). Severe dose-limiting toxicities (e.g. vascular leak syndrome) of systemic IL-2 therapies have prompted the investigation of intra-tumoral and vehicle-driven applications of IL-2 (214). The combination of radiotherapy with the intra-tumoral application of an adenovector encoding IL-12 showed significant tumor regressions with abscopal effects in both subcutaneous and orthotopic hepatoma models. The combination treatment resulted in a reduction of MDSCs, increased functionally activated CD8+ T cells in tumor tissues and enhanced DC maturation (79).

### Ensuring Anti-Tumor Efficacy

#### Inhibition of Immune Checkpoints

Checkpoint inhibitors have substantially shaped the therapy of many malignancies in advanced disease stages, such as malignant melanoma, mismatch repair-deficient colorectal carcinoma and non-small cell lung cancer (215–217). Tumors responsive to checkpoint inhibition have in common a high tumor mutational burden, which directly implicates a high neoantigen burden with immunogenic effects on DCs and T cells (218).

In 2017 and 2018, the FDA granted accelerated approval for Nivolumab and Pembrolizumab in HCC, based on data from the CheckMate 040 and Keynote 224 trials, respectively. Both showed similar response rates of 15-20% (14, 219–221). A more recent development was the approval of the combination of atezolizumab (anti-PD-L1 antibody) and bevacizumab (anti-VEGF antibody) as first-line therapy for patients with unresectable or metastatic HCC, due to its superior efficacy compared to sorafenib in a phase III clinical trial (12).

The observation that only a subset of patients exhibits durable tumor responses to checkpoint inhibition therapy can be explained with the concepts of “cold” and “hot tumors”. “Hot tumors” are characterized by a pre-existing adaptive immune response with CD8+ T cell infiltration, IFN-γ signaling and efficient presentation of tumor antigens. Checkpoint blockade then activates this pre-existing response. Thus, the clinicopathological features of low tumor T cell infiltration, low PD-1 T cell and PD-L1 expression, insufficient neoantigens and low mutational burden as well as the absence of IFN-γ signaling have been linked to a primary resistance to checkpoint inhibition (222). The response to anti-PD1 and anti-CD137 therapy has also been clearly linked to the presence of cross-priming cDC1 (223).

A genomic profiling study from the Barcelona working group noted that approximately 27% of HCCs have a high infiltration of immune cells with respective PD-1 and PD-L1 expression and active IFN-γ signaling (224). The majority of patients in this group showed an active adaptive T-cell response, while the remaining three-quarters of HCC patients did not exhibit positivity for markers predictive of successful checkpoint inhibitor response (224), corroborating the observation from clinical studies, where the response rate of HCC patients to checkpoint inhibition was about 15-20% (13, 14). As such, there is an urgent need to find immunomodulatory treatment options for the remaining majority of patients. Several *in situ* vaccination regimens of *in vivo* HCC models have reported additive effects with checkpoint blockade, e.g. for radiotherapy (80), TLR7/8 agonists (77) and TLR9 agonists (190).

The TME clearly contributes to evasion from checkpoint blockade; for example, TAMs are capable of capturing monoclonal antibodies directed against PD-1 by engaging with the Fc domain, terminating their activating effect on T cells (225). Increased numbers and activity of Tregs can further contribute to an insufficient checkpoint blockade by direct or indirect (production of the anti-inflammatory cytokines IL10 and TGF-β) mechanisms of T cell inhibition (226). In this regard, immunomodulation by *in situ* vaccines is a promising strategy to modulate the TME prior to checkpoint therapy.

## Perspectives and Pitfalls

The primary aim of cancer immunotherapy is to elicit a lasting, durable antitumor immunity based on an effective CD8+ T cell response. Because they harness the entire breadth of TAAs and direct the subsequent immune response, *in situ* vaccines are a highly individual therapy that ideally employs a standardized approach (21). In HCC, there are several disease-specific characteristics that each constitute significant challenges for therapy. These include an elevated risk of recurrence after surgical or locoregional therapy, impaired liver function, chronic hepatic injury and risk of carcinogenesis (227). These specific challenges warrant an intense immunological investigation with a potential to implement *in situ* vaccines here. HCCs typically arise in a fibrous environment and show prominent neovascularization, with a malformed vasculature that inhibits CD8+ T cell infiltration and hampers CD8+ effector functions (228). The underlying liver fibrosis may further impair trafficking of immune cells with impaired antigen recognition due to fibrovascular remodeling (229). Given the clinical efficacy of the Atezolizumab plus Bevacizumab combination and the prominent role of angiogenesis in HCC biology, the exploration of VEGF inhibition to normalize the tumor vasculature may be also warranted for HCC *in situ* vaccination concepts (12, 230). Another challenge in orchestrating a hepatic antitumor response may lie in the inherently tolerogenic direction of hepatic immune responses and hepatic DCs, especially (183). This may be further aggravated by the fact that antigen presentation can also be performed by numerous other hepatic cell types, including liver sinusoidal endothelial cells, hepatocytes, macrophages and Kupffer cells, and contributes to immune tolerance after antigen presentation (231, 232).

As the induction of ICD alone mostly fails to create an effective antitumor response due to insufficient antigen or danger signal release (233), the development of higher-order combination protocols to ensure additional recruitment and activation of APCs as well as the overcoming of the immunosuppressive TME represents the key to success (35). The downside of these approaches might be a more frequent occurrence of immune-related adverse events (irAEs) (234). Although the underlying mechanisms are still not completely understood, the activation of tissue-resident cytotoxic T cells, increased cytokine levels and the formation of auto-antibodies most likely contribute to impaired self-tolerance (235). Since most HCC patients already suffer from chronic inflammatory conditions of the liver, the appearance of liver auto-antigens and activation of CD8+ T cells due to *in situ* vaccination may trigger hepatic irAEs. Although clinical data are still sparse, it has been indicated that HCC patients treated with immune checkpoint inhibitors show higher proportions of hepatic irAEs compared to other treated tumor patients (236). Currently, only limited experimental and clinical evidence is available for *in situ* vaccination in HCC, and the results from upcoming clinical trials are eagerly awaited.

The advent of immunotherapy in multiple solid tumors including HCC has prompted the development of new therapeutic combinations that modulate the TME and the systemic antitumor response. Besides exploring new strategies to optimize the efficacy of standard immunotherapies, it is essential to find approaches that target and guide all essential steps of antitumor immunization. *In situ* vaccines may provide an opportunity to elicit lasting responses against HCC and to overcome the TME.

## Author Contributions

Conceptualization: LH. Investigation: IL, WW, and LH. Writing — Original draft preparation: IL and WW. Writing — Review and Editing: RM, CR, FT, and LH. Supervision: LH. Funding Acquisition: FT. All authors contributed to the article and approved the submitted version.

## Funding

This work was supported by the German Research Foundation (DFG SFB/TRR296, CRC1382, Ta434/3-1 and Ta434/5-1). We acknowledge support from the German Research Foundation (DFG) and the Open Access Publication Funds of Charité—Universitätsmedizin Berlin.

## Conflict of Interest

The authors declare that the research was conducted in the absence of any commercial or financial relationships that could be construed as a potential conflict of interest.

## Abbreviations

ALD, alcohol-related liver disease; APC, antigen-presenting cell; BCG, Bacillus Calmette-Guerin; CAF, cancer-associated fibroblasts; CCL, C-C chemokine ligand; CCR, chemokine receptor; cDC, conventional dendritic cell; CXCL, CXC-ligand; DAMP, damage-associated molecular pattern; DC, dendritic cell; Flt3L, Fms-like tyrosine kinase 3 ligand; FOLFOX, 5-fluorouracil plus oxaliplatin; GM-CSF, granulocyte-macrophage colony-stimulating factor; HCC, hepatocellular carcinoma; HMGB1,high mobility group box 1; HSP, heat shock protein; HSV, herpes simplex virus; ICD, immunogenic cell death; IFN, interferon; IL, interleukin; iNHL, indolent non-Hodgkin lymphoma; irAEs, immune-related adverse events; MDA5, melanoma differentiation-associated gene 5; MDSC, myeloid-derived suppressor cells; MMP, matrix metalloproteinase; mRNA, messenger RNA; NASH, nonalcoholic steatohepatitis; NDV, Newcastle disease virus; NK, Natural Killer; OS, overall survival; PAMP, pathogen associated molecular pattern; PD, programmed death; pDC, plasmacytoid dendritic cell; PD-L, programmed death ligand; polyIC, polyinosinic:polycytidylic acid; PRR, pattern recognition receptor; RAGE, receptor for advanced glycation endproducts; RIG-I, retinoic acid-inducible gene-I; TAA, tumor-associated antigen; TACE, Transarterial Chemoembolization; TAM, tumor-associated macrophage; Th, T helper; TIM-3, T-cell immunoglobulin and mucin-domain containing-3; TLR, toll-like receptor; TME, tumor microenvironment; TNF, tumor necrosis factor; TRAIL, tumor necrosis factor-related apoptosis-inducing ligand; Treg, regulatory T cell; T-VEC, talimogene laherparepvec; VEGF, vascular endothelial growth factor; VSV, vesicular stomatitis virus.
